# Hospital-Acquired Bloodstream Infections in the Adult Intensive Care Unit at Prince Mohammed bin Abdulaziz Hospital, Riyadh, Saudi Arabia

**DOI:** 10.7759/cureus.67158

**Published:** 2024-08-18

**Authors:** Naif S Albudayri, Mohammed Alrowaily, Fatima Rebh, Khalid Alshamarry, Amal Alanazi, Lina Alansari, Muath Almajed, Abdullelah Almutairi, Maha Almutairi

**Affiliations:** 1 Preventive Medicine, Riyadh Third Health Cluster, Riyadh, SAU; 2 Preventive Medicine, Riyadh Second Health Cluster, Riyadh, SAU; 3 Internal Medicine, Prince Mohammed bin Abdulaziz Hospital, Riyadh, SAU; 4 Infection Control, Prince Mohammed bin Abdulaziz Hospital, Riyadh, SAU; 5 Medical Services, Ministry of Interior, Riyadh, SAU; 6 Obstetrics and Gynecology, King Fahad Medical City, Riyadh, SAU

**Keywords:** icu, bloodstream infection, mdro, organism, clabsi

## Abstract

Introduction

Hospital-acquired infections, also called nosocomial infections, are infectious diseases acquired in healthcare facilities at least 48 hours after admission and can't be present at the time of admission. Nosocomial bloodstream infection is a serious medical complication from hospitalization, and it can be potentially preventable by taking certain precautions.

Aim

The aim of this study is to determine the prevalence of central line-related bloodstream infections (CLABSI) with different organisms between January 2022 and February 2024 at the intensive care unit (ICU) at Prince Mohammed bin Abdulaziz Hospital, Riyadh, Saudi Arabia.

Patients and methods

This retrospective cross-sectional study was conducted among ICU adult patients. The data were collected from medical and infection control records. All data for intensive care patients with positive blood cultures, except for the pediatric age group, were collected. Data were tabulated and cleaned in MS Excel, and subsequent data analyses were performed in IBM SPSS Statistics for Windows, Version 26 (Released 2019; IBM Corp., Armonk, New York, United States).

Results

Data from 21 patients were collected and analyzed. The mean age of the participants was 62.9 (SD 15.1) years. Female participants (61.9% (13)) were higher than males (38.1% (8)). All patients were inserted with a non-tunneled central venous catheter (CVC). The mortality rate was 76.2% (16). Vancomycin-resistant enterococci (VRE) was the most commonly detected organism in seven cultures (33.3%), followed by *Candida* species in six cultures (28.6%). *Candida* species were prevalent in younger patients (p=0.021) and those sensitive to medication (p=0.015). Survival analyses between age, gender, and organisms yielded insignificant results (p>0.05).

Conclusion

The major sources of bloodstream infection among adult ICU patients were VRE and *Candida* species. Mortality was common in this population, particularly among patients who were resistant to medication. Hence, strategies to reduce hospital-acquired bloodstream infections are warranted.

## Introduction

Hospital-acquired infections, also called nosocomial infections, are infectious diseases acquired in healthcare facilities at least 48 hours after admission and can't be present at admission. Nosocomial bloodstream infection is a severe medical complication from hospitalization, and it can be potentially preventable by taking certain precautions. Central line-related bloodstream infections (CLABSI), defined as the presence of bacteremia originating from an intravenous catheter, account for 11% of all healthcare-associated infections [1]. The most vulnerable group for nosocomial bloodstream infections are critically ill patients [2,3], which are two to seven times more common in the ICU [4,5] and can account for approximately half of all hospital-acquired BSI [6]. Comorbidities, old age, and indwelling devices increase the occurrence of nosocomial bloodstream infections in the intensive care unit (ICU). CLABSI is one of the leading causes of mortality and morbidity among patients with end-stage renal failure [7]. A single episode of CLABSI can increase hospital stay from 7 to 21 days and healthcare-related financial burden [8].

A prospective observational study conducted between January 2016 and December 2018 in Makkah, Saudi Arabia, reported 20 cases of CLABSI out of 120 hemodialysis patients with male predominance (53%) and a mean age of 60 for all genders. The catheterization duration was significantly longer in patients with CLABSI, with a duration of approximately 11 days among the infected, while in non-infected, it was six days. The most frequent organism culture was *Klebsiella pneumoniae *(35%), *Escherichia coli*, methicillin-resistant *Staphylococcus aureus* (MRSA), *S. aureus*, and Stenotrophomonas, reported 5% for each, and no growth was seen in 45% of CLABSI patients. The overall CLABSI rate was 16.93 per 1000 catheter days [8]. In another local study, data was collected from the years 2011-2016, including all patients from both surgical and medical intensive care units (MICUs). The total number of patients included 34669, of which 67 patients had CLABSI in MICU and 18 patients in SICU. The CLABSI rate was 3.2 per 1,000 central line days over six years of surveillance. The CLABSI cases had a male predominance of 74% and a mean age of 55 years. The primary cause of admission in MICU was cardiovascular diseases (78%), followed by respiratory problems (92%), while in SICU, it was gastroenterology problems (40%) followed by cardiovascular diseases (22%). Laboratory positive culture was seen in 78% of patients and the most frequent comorbidities were hypertension followed by diabetes mellitus. Fever and hypotension were reported in most patients. The most reported organisms *Pseudomonas aeruginosa* (15%), *Candida albicans* (14%), *Candida tropicalis* (11%), *Enterococcus faecium* (11%), extended-spectrum-β-lactamase (ESBL) *K. pneumoniae* (9%), and MRSA (5%) [9]. A Saudi two-year surveillance study including all Ministry of Health hospitals in 2018 and 2019 demonstrated 1542 CLABSI events out of 475913 central line days, which represents a rate of 3.24 per 1,000 cen­tral line days [10]. In addition, a local prospective study stated that CLABSI (25%) was the third most common nosocomial infection in the ICU. It was also reported that *K. pneumoniae* was the most frequent isolate 39 (24%), followed by *Acinetobacter baumannii *35 (21.5%), *P. aeruginosa* 25 (15.3%), and *Proteus *species 23 (14%). In September, most cases occurred, followed by August, July, and May, which reported the same instances. Patients with more than two weeks of ICU stay were associated with an increased risk of CLABSI, and chronic obstructive pulmonary disease (COPD) (50%) was the most common comorbidity [11]. Moreover, a cross-sectional local study reported that gram-negative bacterial organisms were the most frequently isolated from adult and elderly patients (30%). The percentage of gram-positive and negative cultures was 55.9% and 44.01%, respectively. *Staphylococcus epidermidis* (230) was the most common isolates, followed by *K. pneumoniae* (130) and *E. coli *(69) [12].

A prospective surveillance study of 3769 patients hospitalized in four adult ICUs hospitals in four cities in Saudi Arabia reported 4468 central line days and 31 CLABSIs in the baseline periods, accounting for 6.9 CLABSIs per 1000 CL-days. During the intervention, 12027 CL-days and 37 CLABSIs were recorded, accounting for 3.1 CLABSIs per 1000 CL-days. The predominant microorganisms in the baseline and intervention periods were *Acinetobacter *species and *K. pneumonia* [13].

In 2017, 24265 CLABSIs were reported by 3576 United States acute care hospitals to the United States Centers for Disease Control and Prevention's National Healthcare Safety Network [14]. Neutropenic patients are at high risk for CLABSI and hematologic malignancies. Patients with an absolute neutrophil count below 100 cells/mm^3^ appear at the most significant risk [15]. The United States Centers for Disease Control and Prevention reported the distribution of microorganisms in CLABSI as follows: coagulase-negative staphylococci (16.4%), *S. aureus* (13.2%), Enterococci (15.2%), *Candida *species (13.3%), *Klebsiella *species (8.4%) and *E. coli* (5.4%) [16].

In conclusion, central venous catheters are commonly used in critically ill patients; however, central venous catheters (CVCs) have the potential to result in bloodstream infections. Evidence-based guidelines have led to a significant reduction in the incidence of bloodstream infections associated with CVCs. The combination of guideline implementation and newer technologies has the potential to further reduce morbidity and mortality from diseases related to CVCs.

## Materials and methods

Research problem and significance

CLAPSI increases mortality, morbidity, hospital length of stay, and financial burden. The outcome of this study may help public health decision-makers establish policies for CLAPSI prevention. This retrospective cross-sectional study was conducted among ICU adult patients by convenience sampling, and the data were collected from medical and infection control records. The study objectives are to find out the prevalence of CLABSI with different organisms from January 2022 to February 2024 at the ICU at Prince Mohammed bin Abdulaziz Hospital, Riyadh, Saudi Arabia, to explore any associated factors of each organism, to compare different demographic elements, and to study associated comorbidities, reasons for admission, length of hospital stays, and the outcome. All patients with a CLAPSI-positive culture in the ICU from January 2022 to February 2024 will be included in this study. Patients under 18 years old and obstetric cases will be excluded from this study.

Ethical considerations

Prince Mohammed bin Abdulaziz Hospital Institutional Review Board approval was obtained on March 24, 2024. Complete confidentiality of patients' names and medical record numbers (MRNs). All data will be accessed by research participants only.

Statistical analysis

Data were tabulated and cleaned in MS Excel, and all subsequent data analyses were performed in IBM SPSS Statistics for Windows, Version 26 (Released 2019; IBM Corp., Armonk, New York, United States). As appropriate, descriptive statistics were presented using numbers, percentages, means, and standard deviations. The relationship between organisms and the demographic and clinical characteristics of the patients was conducted using the Fischer exact test. Survival analysis was also performed to determine the differences in survival between age, gender, and type of organism in relation to the length of hospital stay. Statistical significance was set to the p<0.05 level.

## Results

This study analyzed 21 patients with a positive culture during the year 2023. About 61.9% were more than 65 years old, with a similar percentage of female patients (61.9%). The most common diagnosis was decompensated heart failure (14.3%), while the most common invasive procedure was central line insertion (33.3%). Fever (33.3%) and hypotension (33.3%) were the most frequent signs and symptoms. All patients received an insertion device; the most common insertion site was jugular (52.4%). Non-tunneled CVC were administered in 19 patients (90.5%). IV medications and fluids were the major indications (81%) (Table 1).

**Table 1:** Demographic and clinical characteristics of the patients (n=21) HD: hemodialysis; MVA: motor vehicle accident; CKD: chronic kidney disease; GCS: Glasgow Coma Scale; CVC: central venous catheter; EGD: esophagogastroduodenoscopy; IV: intravenous

Study variables	N (%)
Age group	
≤65 years	08 (38.1%)
>65 years	13 (61.9%)
Gender	
Male	08 (38.1%)
Female	13 (61.9%)
Diagnosis	
Overload for urgent HD	02 (09.5%)
HD line-related sepsis	02 (09.5%)
COVID-19	02 (09.5%)
MVA	02 (09.5%)
Decompensated heart failure	04 (19.0%)
Cardiac arrest	02 (09.5%)
CKD	02 (09.5%)
GCS	02 (09.5%)
Other	03 (14.3%)
Invasive procedure	
None	04 (19.0%)
Central line insertion	07 (33.3%)
Femoral line insertion	03 (14.3%)
EGD	02 (09.5%)
Surgical debridement	02 (09.5%)
Others	03 (14.3%)
Signs and symptoms	
Fever	07 (33.3%)
Hypotension	07 (33.3%)
Both	05 (23.8%)
Shivering	02 (09.5%)
Device-related	
No	0
Yes	21 (100%)
Site of insertion	
Femolar	07 (33.3%)
Jugular	11 (52.4%)
Other	03 (14.3%)
Type of CVC	
None	02 (09.5%)
Non-tunneled CVC	19 (90.5%)
Indication	
IV meds and fluids	17 (81.0%)
Hemodialysis	03 (14.3%)
Others	01 (04.8%)

**Figure 1:**
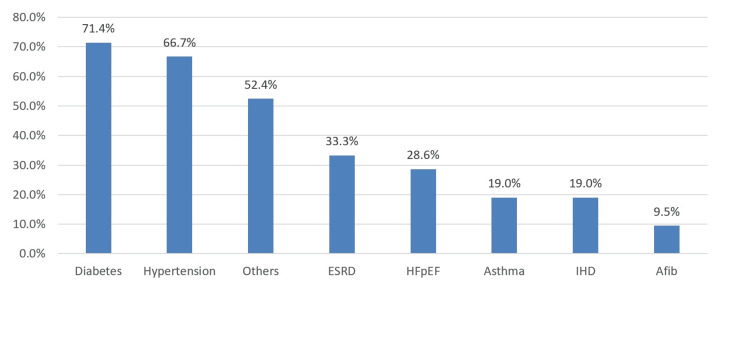
Patient comorbidities ESRD: end-stage renal disease; HFpEF: heart failure with preserved ejection fraction; IHD: ischemic heart disease; Afib: atrial fibrillation

Figure 1 shows that the most commonly associated comorbidity was diabetes (71.4%), followed by hypertension (66.7%) and ESRD (33.3%).

In Table 2, the majority received a meropenem prescription (57.1%), with 1000 mg being the most common dose (55%). The frequency of taking medication is usually once per day (81%). All patients had taken blood specimens. The most commonly detected organism was vancomycin-resistant enterococci (VRE) (33.3%). Resistance to medication was seen in 45%. The mortality rate among CLABSI patients was 76.2%.

**Table 2:** Treatment, organisms, and outcome (n=21) VRE: vancomycin-resistant enterococci

Variables	N (%)
Medication	
Meropenem	12 (57.1%)
Anidulafungin	05 (23.8%)
Bactrim	03 (14.3%)
Ciprofloxacin	01 (04.8%)
Medication dose	
<1000 mg	09 (45.0%)
1000 mg	11 (55.0%)
Frequency	
Once a day	17 (81.0%)
Twice a day	03 (14.3%)
Thrice a day	01 (04.8%)
Blood specimen	
No	0
Yes	21 (100%)
Organism	
VRE	07 (33.3%)
Candida albicans	02 (09.5%)
Klebsiella pneumoniae	02 (09.5%)
Stenotrophomonas maltophilia	02 (09.5%)
Pseudomonas aeruginosa	02 (09.5%)
Serratia marcescens	01 (04.8%)
Enterobacter cloacae	01 (04.8%)
Candida auris	01 (04.8%)
Citrobacter freundii	01 (04.8%)
Candida glabrata	01 (04.8%)
Candida dubliniensis	01 (04.8%)
Sensitivity	
Resistant	11 (55.0%)
Sensitive	09 (45.0%)
Outcome	
Died	16 (76.2%)
Discharged	01 (04.8%)
Transferred	04 (19.0%)

Measuring the relationship between organisms according to the demographic and clinical characteristics of the patients found that patients detected with *Candida *species were more likely to be older (p=0.021) and were sensitive to medications (p=0.015), while patients with VRE were less likely to be hypertensive (p=0.032). No significant relationships were observed between organisms in relation to gender, other comorbidities, invasive procedures, signs and symptoms, site of insertion, medication, and the outcome (p>0.05) (Table 3).

**Table 3:** Relationship between organisms among the demographic and clinical characteristics of the patients (n=21) ^§^: p-value has been calculated using the Fischer exact test; ^**^: significant at p<0.05 level HD: hemodialysis; MVA: motor vehicle accident; CKD: chronic kidney disease; GCS: Glasgow Coma Scale; CVC: central venous catheter; EGD: esophagogastroduodenoscopy; IV: intravenous; ESRD: end-stage renal disease; HFpEF: heart failure with preserved ejection fraction; IHD: ischemic heart disease; Afib: atrial fibrillation; HTN: hypertension; DM: diabetes mellitus; VRE: vancomycin-resistant enterococci

Factor	Organism			p-value^§^
	VRE N (%) (n=7)	*Candida *N (%) (n=6)	Others N (%) (n=8)	
Age group				
≤65 years	02 (28.6%)	05 (83.3%)	01 (12.5%)	0.021^**^
>65 years	05 (71.4%)	01 (16.7%)	07 (87.5%)	
Gender				
Male	03 (42.9%)	01 (16.7%)	04 (50.0%)	0.535
Female	04 (57.1%)	05 (83.3%)	04 (50.0%)	
Comorbidities				
DM	03 (42.9%)	05 (83.3%)	07 (87.5%)	0.189
HTN	02 (28.6%)	05 (83.3%)	07 (87.5%)	0.032^**^
Asthma	01 (14.3%)	01 (16.7%)	02 (25.0%)	1.000
ESRD	01 (14.3%)	03 (50.0%)	03 (37.5%)	0.444
IHD	02 (28.6%)	0	02 (25.0%)	0.495
Afib	01 (14.3%)	0	01 (12.5%)	1.000
HFpEF	03 (42.9%)	01 (16.7%)	02 (25.0%)	0.707
Invasive procedure				
None	02 (28.6%)	01 (16.7%)	01 (12.5%)	0.184
Central Line insertion	04 (57.1%)	0	03 (37.5%)	
Femoral line insertion	01 (14.3%)	0	02 (25.0%)	
EGD	0	02 (33.3%)	0	
Surgical debridement	0	01 (16.7%)	01 (12.5%)	
Others	0	02 (33.3%)	01 (12.5%)	
Signs and symptoms				
Fever	02 (28.6%)	03 (50.0%)	02 (25.0%)	0.359
Hypotension	03 (42.9%)	03 (50.0%)	01 (12.5%)	
Both	02 (28.6%)	0	03 (37.5%)	
Shivering	0	0	02 (25.0%)	
Site of insertion				
Femolar	02 (28.6%)	03 (50.0%)	02 (25.0%)	0.622
Jugular	05 (71.4%)	02 (33.3%)	04 (50.0%)	
Other	0	01 (16.7%)	02 (25.0%)	
Medication				
Meropenem	05 (71.4%)	02 (33.3%)	05 (62.5%)	0.076
Anidulafungin	01 (14.3%)	04 (66.7%)	0	
Bactrim	01 (14.3%)	0	02 (25.0%)	
Ciprofloxacin	0	0	01 (12.5%)	
Sensitivity				
Resistant	06 (85.7%)	0	05 (62.5%)	0.015^**^
Sensitive	01 (14.3%)	05 (100%)	03 (37.5%)	
Outcome				
Died	06 (85.7%)	04 (66.7%)	06 (75.0%)	0.827
Alive	01 (14.3%)	02 (33.3%)	02 (25.0%)	

**Figure 2:**
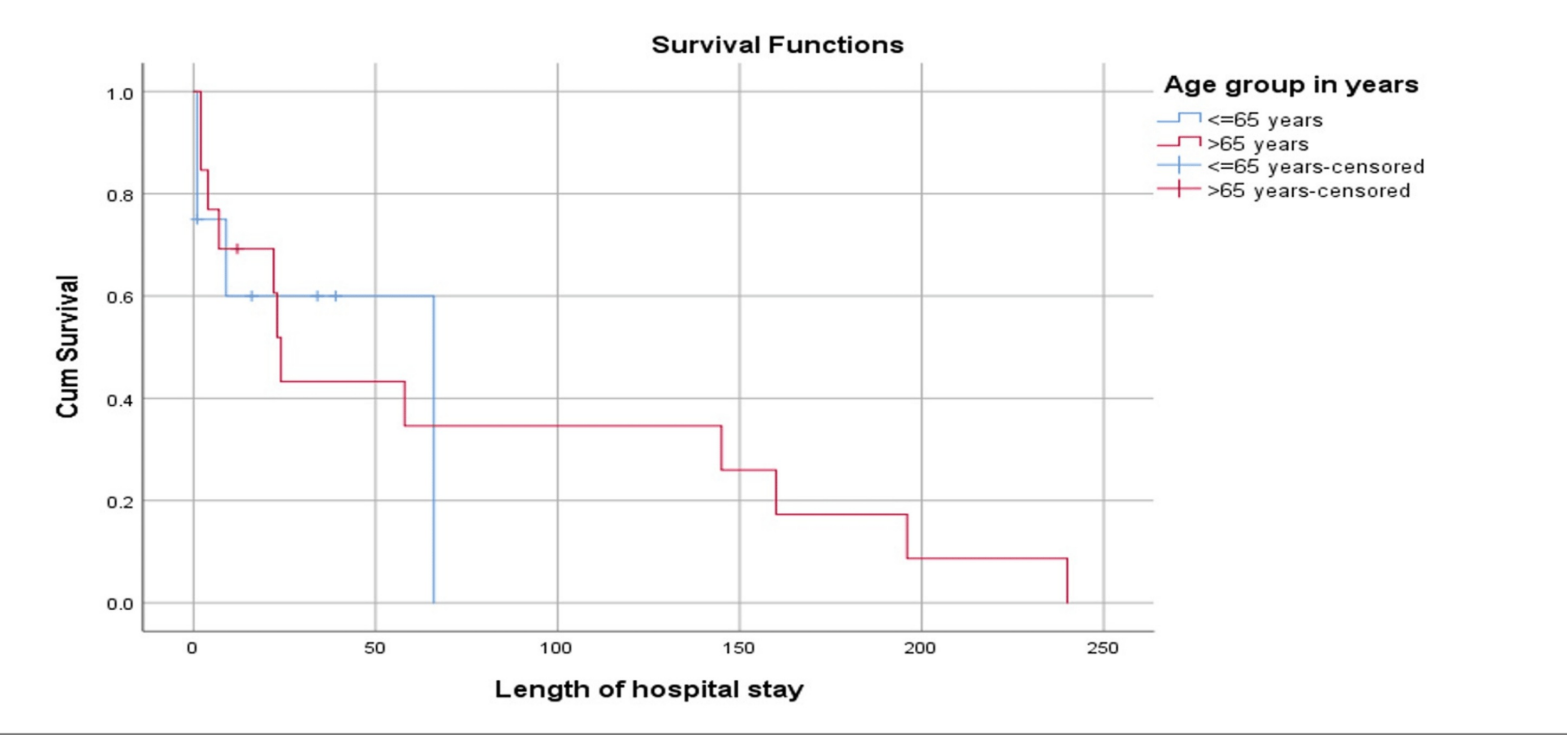
Survival plot according to age group

Figure 2 illustrates the survival plot between age groups (≤65 years vs. >65 years) in relation to the length of hospital stay. According to the results, the mean survival time for the age group ≤65 years was 41.2 (standard (STD) error: 12.9) days, while the mean survival time for the age group >65 years was 76.3 (STD error: 25.2) days, and the overall mean survival time was 73.9 (STD error: 20.8) days. Analysis revealed that based on Log-rank Mantel-cox, the difference was not statistically significant (p=0.794).

**Figure 3:**
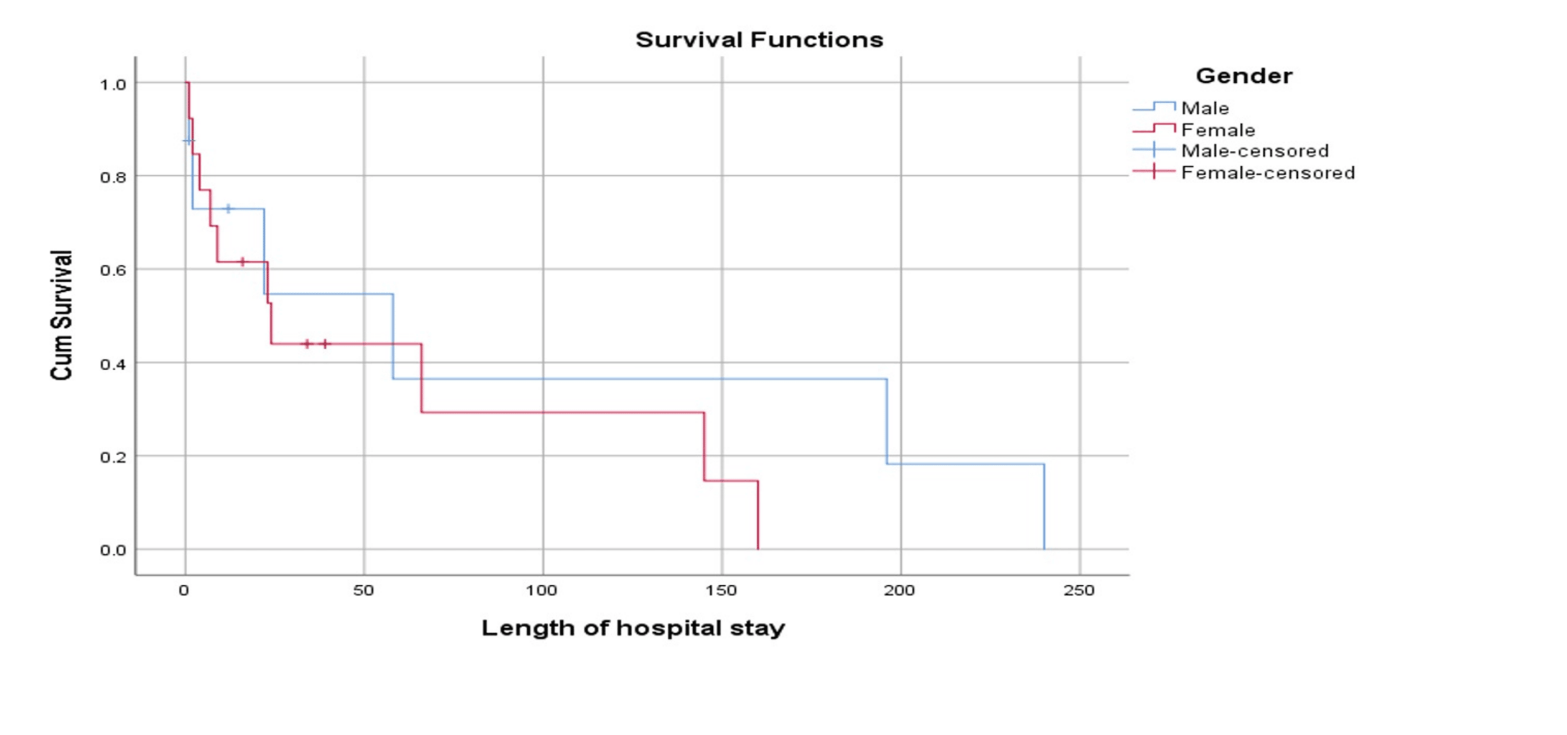
Survival plot according to gender

Figure 3 depicts the survival plot between males and females in relation to the duration of hospital stay. It can be observed that the mean survival time for males was 94.5 (STD error: 43.1) days. The mean survival time for females was 60.3 (STD error: 20.3) days, and the overall mean survival time was 73.9 (STD error: 20.8) days. Analysis revealed that based on Log-rank Mantel-cox, the difference was not statistically significant (p=0.400).

**Figure 4:**
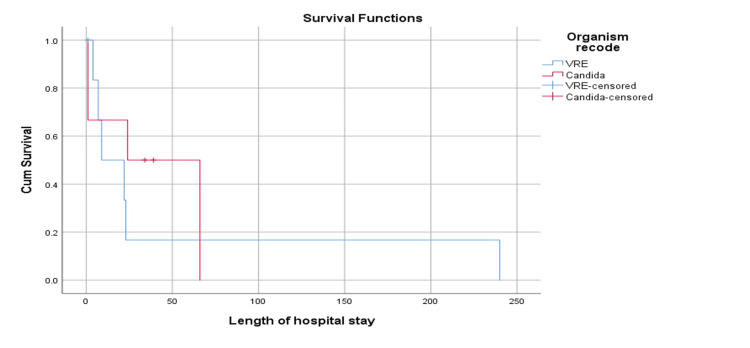
Survival plot according to organism VRE: vancomycin-resistant enterococci

Figure 4 shows the survival plot between patients with VRE and patients with *Candida *species concerning the duration of hospital stays. It was revealed that the mean survival time for VRE was 50.8 (STD error: 37.9) days, while the mean survival time for *Candida *was 37.3 (STD error: 13.9) days. Overall mean survival time was 54.5 (STD error: 30.0) days. Analysis revealed that based on Log-rank Mantel-cox, the difference was not statistically significant (p=0.575).

When examining the relationship between the outcome and the sensitivity, it was found that 66.7% of patients who were resistant to medication had died, but this difference did not reach statistical significance (p=0.127) (Table 4).

**Table 4:** Relationship between outcome and sensitivity (n=21) ^§^: p-value has been calculated using the Fischer exact test; ^**^: significant at p<0.05 level

Factor	Outcome		p-value^§^
	Died N (%) (n=16)	Alive N (%) (n=5)	
Sensitivity			
Resistant	10 (66.7%)	01 (20.0%)	0.127
Sensitive	05 (33.3%)	04 (80.0%)	

## Discussion

The CLABSI monitoring and prevention program had a significant role in reducing CLABI rates. This study investigated 21 patients who developed CLABSI during ICU admission. Out of these, seven patients (33.3%) were detected to have VRE, six patients (28.6%) had *Candida *species (i.e., *C. albicans*, *Candida auris*, etc.), and the rest had a few cases of *K. pneumonaie* (two cases), *Stenotrophomonas maltophilia* (two cases), *P. aeruginosa* (two cases), *Serratia marcescens* (one case), and *Enterobacter cloacae* (one case). Studies across Saudi Arabia provided conflicting reports regarding bloodstream infections in critical care units. For example, among 20 patients who developed blood infections in Makkah, *K. pneumonaie *was the most common isolated organism being discovered; the two-year incidence rate was 24.06 per 100 catheter days [8]. Another study conducted in Riyadh found that over six years, the CLABSI rate was 3.2 per 1,000 central line days (CL-days), with *P. aeruginosa *followed by *C. albicans *being the most frequently identified microorganisms that caused bloodstream infection in the MICU. Further, poly-microorganism cases were more prominent in MICU (90%) than in the surgical intensive care unit (SICU) (10%) [9]. In the UK, a study documented that the most frequent causes of bloodstream infections among cirrhotic patients were *Klebsiella *species, coagulase-negative staphylococci, and *E. faecium *[16]. Patients admitted to the ICU are at greater risk for secondary bloodstream infection. Hence, continuous surveillance is important to reduce the risk of healthcare-associated infections (HAIs) among critically ill patients.

Data from our study suggest that younger patients (≤65 years) and those sensitive to medication were at increased risk of developing bloodstream infections, particularly *Candida *species. However, patients with hypertension were more likely to develop other microorganisms (e.g., *K. pneumonaie*) but not VRE. This is not consistent with the study of Mollee et al. (2011) [14]. According to their reports, catheter-associated bloodstream infection (CABSI) varies significantly according to the type of central venous access devices (CVADs), patient diagnosis, side of insertion, and the frequency of prior line insertions. However, previous reports by Despotovic et al. (2020) documented different scenarios as the variations of HAI acquisition differed significantly by the underlying viral central nervous system (CNS) infections and invasive devices (urinary and central venous catheters and nasogastric tubes) [17].

Mortality rates among adult ICU patients who developed CLABSI were common. Based on our results, among 21 subjects, 16 had died, giving an overall one-year mortality rate of 76.2%. However, mortality rates yielded no difference when compared to CLABSI type (p=0.827). Contradicting these reports, Harte et al. (2021) showed a lower incidence rate of mortality among patients with bacteremia at 33% [18]. Notwithstanding these reports, Wozniak et al. (2024) found that an increased risk of mortality was associated with cirrhosis diagnosis, which may have been due to the high prevalence of *E. faecium* bloodstream infection [16].

There was no difference in survival rates between patients who developed VRE and *Candida *species (p=0.575). The mean survival time of VRE patients was 50.8 days, while that of *Candida *patients was 37.3 days. Further, we did not find significant variations in survival time between gender and age in relation to the duration of hospital stay (p>0.05). The small sample in our study could influence the outcome and warrant further investigations. A study conducted in Kenya showed that the chance of survival among CLABSI patients is greater than in patients without CLABSI. However, this scenario was inconsistent at about 113 days, as greater survival probabilities shifted to non-infected patients. During 140 days of admission, there was a steep decline in the survival probabilities of the infected group [19].

Regarding CLABSI treatment, meropenem and anidulafungin were the most frequently used medications. Further, resistance to medications was seen in more than half of CLABSI patients; however, we found no association between mortality rates and sensitivity or resistance to medications (p=0.127). This is in agreement with the study done in Serbia [17]. Resistant rates were identified in over 50% of cases and were seen in almost all antimicrobial drugs, with the exception of colistin and tigecycline. Among 1039 patients with bacterial isolates in Riyadh [11], gram-negative isolates were resistant to cephalothin, amoxicillin-clavulanate, and ampicillin while sensitive to ertapenem, amikacin, trimethoprim-sulfamethoxazole, and colistin. On the other hand, gram-positive isolates were resistant to amoxicillin-clavulanate, oxacillin, and cephalothin but were sensitive to gentamicin, penicillin, vancomycin, and ciprofloxacin.

This study has potential limitations. A small sample size can influence the results of this study. A good way to fix it is to increase the time of the study or do a multicenter approach in the same city to create a larger sample size. Time constraints were an issue that prevented us from increasing the time and collecting more data.

## Conclusions

VRE and *Candida *species were the major sources of hospital-acquired bloodstream infections among adult patients admitted to the ICU in Prince Mohammed bin Abdulaziz Hospital. Patients with *Candida *species were more likely to be younger and were more sensitive to medication. Further, one-third had central line insertion, and non-tunneled CVC was the main type of CVC used. Interestingly, there was no difference in the patient's survival time in terms of age, gender, or organism in relation to the duration of the hospital stay. Efforts to reduce the risk of infection in intensive care settings are necessary, with a focus on patients who have undergone invasive procedures.
